# Epidemiological impacts of post-infection mortality

**DOI:** 10.1098/rspb.2023.0343

**Published:** 2023-07-12

**Authors:** Chadi M. Saad-Roy, Simon A. Levin, Bryan T. Grenfell, Mike Boots

**Affiliations:** ^1^ Miller Institute for Basic Research in Science, University of California, Berkeley, CA, USA; ^2^ Department of Integrative Biology, University of California, Berkeley, CA, USA; ^3^ Department of Ecology and Evolutionary Biology, Princeton University, Princeton, NJ, USA; ^4^ School of Public and International Affairs, Princeton University, Princeton, NJ, USA; ^5^ Department of Biosciences, University of Exeter, Penryn, UK

**Keywords:** post-infection mortality, epidemiological model, periodicity

## Abstract

Infectious diseases may cause some long-term damage to their host, leading to elevated mortality even after recovery. Mortality due to complications from so-called ‘long COVID’ is a stark illustration of this potential, but the impacts of such post-infection mortality (PIM) on epidemic dynamics are not known. Using an epidemiological model that incorporates PIM, we examine the importance of this effect. We find that in contrast to mortality during infection, PIM can induce epidemic cycling. The effect is due to interference between elevated mortality and reinfection through the previously infected susceptible pool. In particular, robust immunity (via decreased susceptibility to reinfection) reduces the likelihood of cycling; on the other hand, disease-induced mortality can interact with weak PIM to generate periodicity. In the absence of PIM, we prove that the unique endemic equilibrium is stable and therefore our key result is that PIM is an overlooked phenomenon that is likely to be destabilizing. Overall, given potentially widespread effects, our findings highlight the importance of characterizing heterogeneity in susceptibility (via both PIM and robustness of host immunity) for accurate epidemiological predictions. In particular, for diseases without robust immunity, such as SARS-CoV-2, PIM may underlie complex epidemiological dynamics especially in the context of seasonal forcing.

## 1. Introduction

The ongoing SARS-CoV-2 pandemic starkly illustrates the potential of infectious diseases to continue to cause significant infections and mortality across the world. It has also focused attention on the importance of pathogen-induced mortality after active infection (e.g. [1–5]), i.e. post-infection mortality (hereafter referred to as ‘PIM’). Thus, to understand the potential future dynamics of SARS-CoV-2, it is important to characterize the effects of PIM on epidemic dynamics. In a broader context, this current observation of PIM for SARS-CoV-2 is part of a wider body of work that examines the long-term impact of infections on hosts. For example, historical findings suggest elevated later-life mortality due to airborne pathogens encountered early in life [6]. Additionally, studies link inflammation due to infectious diseases (early in life) with an increase in later-life mortality [7,8]. Despite these effects being widespread, epidemiological models typically only include virulence during the infectious stage and ignore PIM.

In general, PIM can emerge via (at least) two biological mechanisms. First, pathogens may cause damage that does not lead to host death during active infection, but that eventually results in other impacts that shorten host lifespan (compared to never-infected susceptible individuals). For instance, a number of other long-term complications from SARS-CoV-2 infections, such as cardiovascular issues [9], indicate potential mechanisms for increased mortality following recovery. Other examples of pathogens that result in long-term effects following recovery from infection range from human papilloma virus (HPV) causing cancer [10], to Epstein–Barr virus potentially causing multiple sclerosis [11]. Additionally, recent work has shown that immunomodulation due to measles infection increases subsequent susceptibility to other diseases [12,13]. In very recent work, Levine *et al.* [14] examined the impact of multiple viruses on neurodegeneration and found multiple positive associations. In turn, these consequences increase the likelihood of PIM.

Second, to successfully clear an infecting pathogen, hosts may disproportionately allocate resources to mount a robust immune response. This could then lead to other consequences that increase mortality after infection, in contrast to those susceptible individuals that have never been infected. For example, a parasitic nematode infection can trigger the release of interleukin 33 (IL-33) as a defence mechanism [15]; in turn, IL-33 might contribute to inflammatory bowel disease [16], which might lead to a decreased lifespan [17]. Related trade-offs have also been invoked to understand the evolutionary emergence of immune systems (e.g. [18]). Both of these mechanisms are very general and there is therefore considerable potential for PIM to be the common outcome of infection.

While there exists a significant body of epidemiological modelling literature that includes host mortality during active infection (often referred to as ‘virulence’) (see, e.g. [19–22]) dynamic impacts of PIM has generally been overlooked. Busenberg & van den Driessche [23] formulated and analysed a model with additional mortality in the infectious and recovered (fully immune) states, where individuals return to complete susceptibility with no additional mortality once host immunity has waned. However, the epidemiological dynamics that result from PIM coupled with a return to (potentially partial) susceptibility is unknown. Recent models that include buffered susceptibility have shown that the strength of immunity can crucially shape epidemic dynamics [24–27]. In particular, simple epidemiological models have revealed that the relative susceptibility to subsequent infection (once a period of complete natural or vaccinal immunity has waned) can govern medium-term epidemic trajectories [25]. Therefore, a key question is to determine the epidemiological impacts of potential interactions between PIM and host immunity via a reduced susceptibility to reinfection.

Here, we study the impact of PIM on epidemic dynamics. We also examine the interplay that emerges if either host immune responses (that reduce the likelihood of reinfection upon recovery) are present or if there is disease-induced mortality during active infection. To accomplish this, we leverage a simple model embedded with PIM, buffered susceptibility and disease-induced mortality during active infection. We show that PIM has strong destabilizing effects both on its own and in combination with virulence during infection unless acquired immunity is very robust.

## 2. Model framework

**Figure 1.**
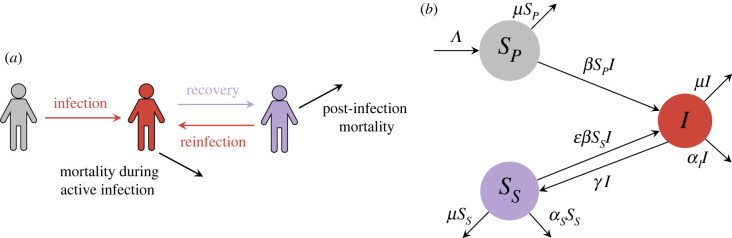
Formulation of epidemiological model with PIM. (*a*) Schematic illustration of epidemiological processes that are encompassed in the model framework. (*b*) Model flow diagram with rates into/out of each compartment.

To distill the effect of PIM on epidemiological dynamics, we use a mathematical model that distinguishes between never-infected susceptibles and those susceptible individuals that were previously infected (figure 1*a*).

In our model, recruited individuals (via birth and immigration, at constant rate Λ) are first never-infected (and fully) susceptible (*S*_*P*_). By successful infection, they become infectious (*I*), and recover to the ‘secondary’ susceptible class (*S*_*S*_) at rate *γ*. In this stage, PIM occurs at rate *α*_*S*_. After recovery, individuals’ relative susceptibility to (re)infection is ε, where 0≤ε≤1. Thus, if infection confers some immunity against reinfection, then ε<1. On the other hand, if immune responses are absent after recovery, then ε=1. Furthermore, *α*_*I*_ denotes the rate of infection-induced mortality during active infection. Finally, *μ* denotes the rate of demographic death. Our model is depicted in figure 1*b* and formulated as follows:

2.1a $$\frac{dS_{P}}{dt}=\Lambda -\beta S_{P}I-\mu S_{P}$$

2.1b $$\frac{dI}{dt}=\beta I(S_{P}+\varepsilon S_{S})-(\gamma +\mu +\alpha_{I})I$$

2.1c $$\mathrm{and}\;\frac{dS_{S}}{dt}=\gamma I-\varepsilon \beta S_{S}I-\mu S_{S}-\alpha_{S}S_{S}.\;$$

Since the total population *N* = *S*_*P*_ + *I* + *S*_*S*_, it follows that

2.2 $$\frac{dN}{dt}=\Lambda -\mu N-\alpha_{I}I-\alpha_{S}S_{S},$$

and any three of the *S*_*P*_, *I*, *S*_*S*_ and *N* equations determine the dynamics in this system.

## 3. Epidemiological dynamics

In Model (2.1), it is clear that there is a disease-free equilibrium *P*_0_ with $S_{P,0}=\Lambda /\mu$ and *I*_0_ = *S*_*S*,0_ = 0. Since there is only one infected compartment, rearranging $(\partial /\partial I)(dI/dt){|}_{P_{0}}>0$ gives that the basic reproduction number $R_{0}$ is

3.1 $$R_{0}=\frac{\beta (\Lambda /\mu )}{\gamma +\mu +\alpha_{I}}.$$

Biologically, since Λ/μ is the size of the population when there is no disease, β(Λ/μ) represents the number of new infections per time in a fully susceptible population. Furthermore, 1/(*γ* + *α*_*I*_ + *μ*) is the average time an infectious individual spends in the *I* class. Thus, $\beta (\Lambda /\mu )/(\gamma +\mu +\alpha_{I})$ is the average number of new infections in a fully susceptible population. Note that $R_{0}$ in this model does not depend on PIM (*α*_*S*_).

Intuitively, and as generally with compartmental models in epidemiology (see [28,29]), $R_{0}=1$ is a key epidemiological threshold. If $R_{0}<1$, it follows that *P*_0_ is locally asymptotically stable [29]. On the other hand, if $R_{0}>1$, *P*_0_ is unstable and there is a unique endemic equilibrium $\hat{P}=({\hat{S}}_{P},\hat{I},{\hat{S}}_{S})$ (theorem 1; electronic supplementary material).

### (a) Endemic equilibrium characteristics

Explicit formulas for the endemic values of ${\hat{S}}_{P}$, $\hat{I}$ and ${\hat{S}}_{S}$ are given in the electronic supplementary material (see proof of theorem 1; electronic supplementary material). To guide our understanding of how PIM affects epidemic dynamics, we examine how the endemic states in our model vary with PIM. We find that the equilibrium value of never-infected susceptibles, ${\hat{S}}_{P}$, is an increasing function of PIM (theorem 2; electronic supplementary material). By contrast, the equilibrium values of infectious and previously infected susceptible individuals, $\hat{I}$ and ${\hat{S}}_{S}$, decrease as PIM increases (theorem 2; electronic supplementary material).

In electronic supplementary material, figure S1, we illustrate how ${\hat{S}}_{P}$, $\hat{I}$ and ${\hat{S}}_{S}$ change as *α*_*S*_ increases, for different robustness of immunity ε (electronic supplementary material, figure S1A–S1C), transmission rate (electronic supplementary material, figure S1D–S1F) and disease-induced mortality during active infection (electronic supplementary material, figure S1G–S1I). Across scenarios, ${\hat{S}}_{P}$ increases and eventually decelerates as PIM increases (electronic supplementary material, figure S1A, S1D and S1G). In tandem, while $\hat{I}$ decreases when PIM increases, it seems to eventually also decelerate (electronic supplementary material, figure S1B, S1E and S1H).

The impact of other host and pathogen characteristics on the dependence of ${\hat{S}}_{S}$ on PIM is less clear, as illustrated by comparisons within and across additional disease parameters (electronic supplementary material, figure S1C, S1F and S1I). Thus, the landscape of susceptibility at the population level crucially depends on the degree of PIM. Additionally, how this susceptibility landscape changes as PIM increases is determined by the robustness of immunity, the level of pathogen transmission and the degree of disease-induced mortality during active infection.

In the absence of PIM, i.e. *α*_*S*_ = 0, the endemic equilibrium in Model (2.1) is always locally asymptotically stable whenever it exists (theorem 3; electronic supplementary material). Therefore, in the long term, epidemiological trajectories attain this value.

### (b) Post-infection mortality can induce epidemic cycles

If PIM occurs, i.e. *α*_*S*_ > 0, determining the stability of the endemic equilibrium is more complicated, and the eigenvalues of the Jacobian matrix for the *S*_*S*_, *I* and *N* equations at the endemic equilibrium can be numerically computed (see remark 1; electronic supplementary material for the Jacobian matrix). To titrate the effect of PIM on epidemic dynamics, we begin our analyses by examining the limiting case with no immunity following recovery, i.e. ε=1, akin to an SIS model. (In particular, if there is no PIM [i.e. *α*_*S*_ = 0], our model reduces to an SIS model [by setting *S* = *S*_*P*_ + *S*_*S*_].) We also first assume that there is no mortality during active infection (*α*_*I*_ = 0). For other parameters, we take the recovery rate $\gamma =1\;{\mathrm{week}}^{-1}$, i.e. the infection lasts a week, and $\mu =0.02\;{\mathrm{year}}^{-1}$. Finally, we assume that the magnitude of the recruitment rate Λ is equal to that of *μ*. For interpretation, note that this last assumption means that the state variables *S*_*P*_(*t*), *I*(*t*), *S*_*S*_(*t*) and *N*(*t*) therefore denote fractions of the population in these classes relative to the initial (disease-free) population size. Thus, the transmission rate *β* is approximately equal to $R_{0}$ in magnitude.

**Figure 2.**
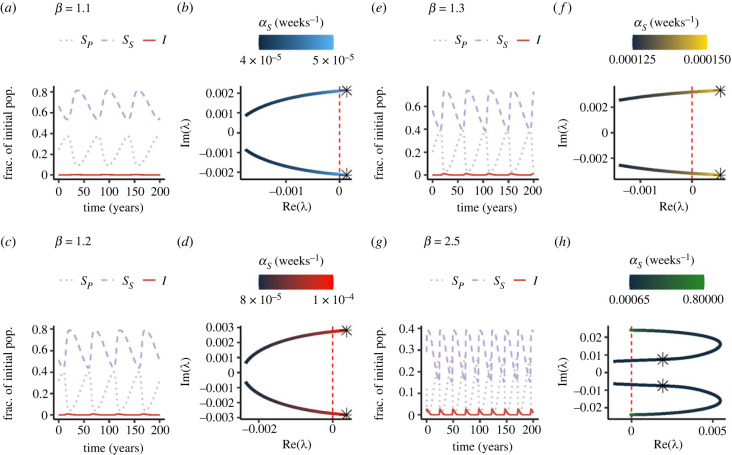
Illustrative examples of time series of *S*_*P*_(*t*), *I*(*t*) and *S*_*S*_(*t*) for the periodic behaviour that can arise due to PIM, for different transmission rates *β* and different PIM rates *α*_*S*_. Since *γ* = 1 and $\Lambda =\mu =\frac{1}{50(52)}$, $R_{0}=\beta /\left(1+\mu \right)\approx \beta$. For (*a*,*c*,*e*) and (*g*), we simulate 600 years with the first week having *I*(*t*_0_) = 10^−9^, *S*_*P*_(*t*_0_) = 1 − *I*(*t*_0_) and *S*_*S*_(*t*_0_) = 0, and we plot weeks 400(52) + 1 to 600(52). Panels (*b*,*d*,*f*) and (*h*) present the values of the complex-conjugate pair of eigenvalues of the Jacobian matrix of the *S*_*S*_, *I* and *N* equations about the endemic equilibrium, for different values of PIM *α*_*S*_. The ‘star’ symbol in each of these plots corresponds to the value of *α*_*S*_ used for the corresponding previous plot. In all four panels, *α*_*I*_ = 0 and ε=1.

Figure 2 illustrates potential epidemiological dynamics with PIM for realistic parameter values. As PIM increases, we find that epidemic cycles can appear. As a function of the rate of PIM and for different transmission rates, we plot in figure 2*b*,*d*,*f*,*h* a pair of eigenvalues (in the complex plane) of the Jacobian matrix (remark 1; electronic supplementary material) whose real parts become eventually positive with large enough PIM (*α*_*S*_ indicated by the line colour). Since the real parts of a pair of eigenvalues become positive, there is a Hopf bifurcation that gives rise to a limit cycle. Note that, for each plot, we have selected these values of PIM to highlight that PIM can lead to oscillations. In figure 2*a*,*c*,*e*,*g*, we present corresponding illustrative time series of the epidemiological dynamics for increasing transmission rates with sufficient PIM to trigger periodicity (each star symbol in figure 2*b*,*d*,*f*,*h* illustrates the value of PIM used for the time series in the previous panel). In these figures, to illustrate the limit cycle and because the cycle period can be long, we plot weeks (52 × 400) + 1 to 52 × 600.

As highlighted by figure 2, low values of PIM are sufficient to trigger periodicity, particularly for low $R_{0}$. As the transmission rate increases in the examples of figure 2, larger values of PIM are necessary for epidemic cycling; and the resulting cycles have shorter periods. If PIM is very strong, the real part of the pair of eigenvalues becomes negative again (figure 2*h*). Intuitively, very high PIM means that the contribution of previously infected individuals to the susceptible pool is substantially reduced and eventually is negligible. In the limit of *α*_*S*_ → ∞, i.e. individuals die immediately upon ‘recovery’, our model is akin to an SI epidemiological model. In an SI model, the endemic equilibrium is locally asymptotically stable whenever it exists.

**Figure 3.**
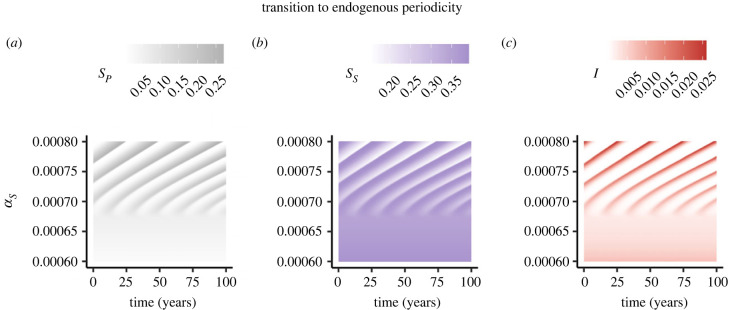
Illustrative examples of PIM triggering endogenous periodicity. Heat maps of time series of (*a*) *S*_*P*_, (*b*) *S*_*S*_ and (*c*) *I* as PIM is varied. In all panels, *β* = 2.5, *γ* = 1, *α*_*I*_ = 0, $\mu =\frac{1}{50(52)}$ and ε=1. To obtain these heat maps, we simulate 500 years with initial conditions (at the first week) of *I*(*t*_0_) = 10^−9^, *S*_*P*_(*t*_0_) = 1 − *I*(*t*_0_) and *S*_*S*_(*t*_0_) = 0. For the heat maps, we discard the first 400 years and plot weeks 400(52) + 1 to 500(52).

In figure 3, we illustrate the transition from a stable endemic equilibrium to periodicity as PIM increases. Since the resulting cycle is shorter for these parameter values than for those in figure 2*a*,*c*,*e*, we plot weeks (52 × 400) + 1 to 52 × 500 instead. As seen in figure 3*c*, the periodic behaviour can lead to large changes in *I*(*t*) compared with the endemic equilibrium values for smaller values of *α*_*S*_ that do not lead to cycles. Interestingly, the infection peak of the cycle appears to be higher for larger PIM (figure 3*c*). In electronic supplementary material, figure S2, we also highlight a limit cycle example in the *S*_*S*_–*S*_*P*_ phase plane (note that in electronic supplementary material, figure S2, we plot weeks (52 × 300) + 1 to 52 × 500 to illustrate the behaviour in the *S*_*S*_–*S*_*P*_ phase plane prior to (and including) that visualized in figure 3).

Since disease transmission can often be seasonal, we examine in electronic supplementary material, figure S3 the impact of an annually forced transmission rate, i.e. *β*(*t*) = *β*_0_(1 + *δ*sin (2*πt*/52)). When PIM is weak and the endemic equilibrium is stable, seasonal variations in transmission lead to annual cycles (e.g. electronic supplementary material, figure S3A). However, when PIM enables cycles, we find that adding a seasonal transmission rate can lead to complex dynamics (electronic supplementary material, figure S3B–S3D). In particular, small outbreaks are first immediately followed by larger ones; these then abate, slowly decreasing to very low levels of infection. During this time, the susceptible pool builds up, thus enabling this process to repeat.

### (c) Impacts of other characteristics on epidemic dynamics with post-infection mortality

To determine the impact of PIM on epidemic dynamics, we have focused on the simplest SIS-like model that includes PIM but no disease-induced mortality during active infection nor immunity following recovery. However, directly transmitted diseases often deviate from these basic assumptions, and increased model complexity can lead to subtle effects on epidemic dynamics. In particular, these additional biological complexities can interplay with the emergent periodicity driven by PIM. We illustrate these with examples below.

#### (i) Host immunity

**Figure 4.**
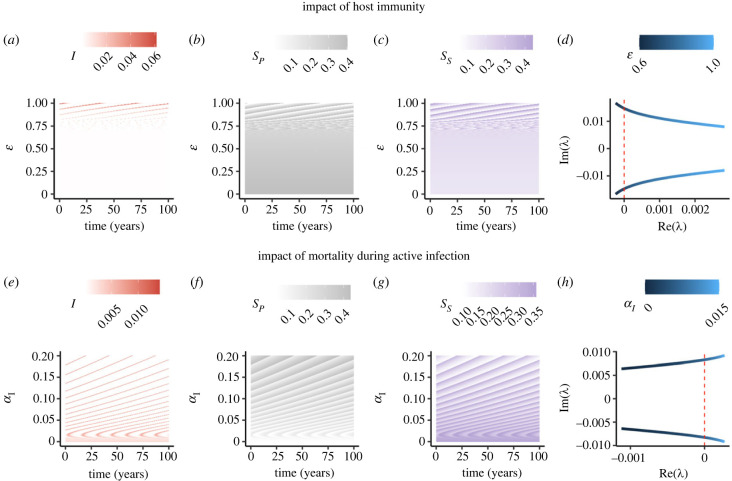
Interplay of additional host and pathogen characteristics with PIM. (*a*–*d*) Illustrative example for the impact of host immunity on epidemic dynamics with PIM. The heat maps in (*a*–*c*) are as in figure 3, but with ε varying instead of *α*_*S*_. (*d*) As in figure 2*b*,*d*,*f*,*h*, but with ε varying. In (*a*–*d*), *β* = 2.5, *α*_*S*_ = 0.001, *γ* = 1, *α*_*I*_ = 0 and $\mu =\frac{1}{50(52)}$. (*e*–*h*) Illustrative example for the impact of disease-induced mortality during active infection. Panels (*e*–*g*) are as in figure 3, but with *α*_*I*_ varying instead of *α*_*S*_. (*h*) As in that of (*d*), but with *α*_*I*_ varying instead of ε. In (*e*–*h*), *β* = 2.5, ε=1, *α*_*S*_ = 0.00065, $\mu =\frac{1}{50(52)}$, *γ* = 1 and $\mu =\frac{1}{50(52)}$. For the heat maps of (*a*–*c*) and (*e*–*g*), and as in figure 3*a*, weeks 400(52) + 1 to 500(52) are plotted.

Many diseases do not exhibit classic SIS-type characteristics, with hosts developing some immunity following recovery. For example, measles elicits very robust immunity following recovery [30]. Other pathogens, such as respiratory syncytial virus or rotavirus, elicit immunity against reinfection, but it is incomplete [31,32]. In these cases, ε<1, i.e. the relative susceptibility to reinfection is *less* than to primary infection. We find that with partial immunity, periodicity can still occur with PIM (figure 4*a*–*d*).

In the limiting case where host immunity following infection is ‘perfect’ and no reinfection is possible, i.e. ε=0, then the endemic equilibrium $\hat{E}$ is locally asymptotically stable (theorem 4; electronic supplementary material). This result implies that for a fixed value of PIM that induces cycling in the SIS case, robust enough host immunity catalyses a collapse of the epidemic cycle to the endemic equilibrium. We illustrate this transition in figure 4*a*–*d*. Intuitively, as immunity increases and the critical ‘collapse’ point approaches, more frequent epidemics occur (figure 4*a*), and the landscapes of susceptibility correspondingly change (figure 4*b*,*c*). If PIM is higher, then periodicity can persist with stronger immunity, i.e. for smaller values of ε (see electronic supplementary material, figure S4).

#### (ii) Pathogen-induced mortality during active infection

So far, we have focused on settings where there is no pathogen-induced mortality during active infection, i.e. *α*_*I*_ = 0. However, pathogens can cause some infectious individuals to die during infection (see e.g. [19]). Therefore, we next relax this initial assumption and examine the impact of death due to disease during infection, i.e. *α*_*I*_ > 0, on epidemic dynamics with PIM.

When there is no PIM, we have proved that the endemic equilibrium is locally asymptotically stable if $R_{0}>1$ (theorem 3; electronic supplementary material). Surprisingly, we find that, in the presence of some limited PIM not sufficiently strong enough to trigger cycling by itself, disease-induced mortality during active infection can cause a transition from a stable endemic state to periodicity (figure 4*e*–*h*). Thus, this analysis illustrates that disease-induced death during infection can heighten the impact of PIM on epidemic dynamics.

## 4. Discussion and conclusion

Finding periodicity in simple epidemiological models has long been a research focus (e.g. [33]). For example, with nonlinear incidence rates to model transmission, Liu *et al.* [34,35] found that a variety of behaviours are possible, including periodic solutions. In other work, Hethcote *et al.* [36] proved that periodicity can occur in SIRS models with three or more recovered classes. In a generalization of this result, Röst & Tekeli [37] showed that cycles can emerge (via Hopf bifurcation) in SIS models with four or more infected stages. Furthermore, in a simple mathematical model, Tidbury *et al.* [38] found that immune priming can cause periodicity. By contrast, Busenberg & van den Driessche [23] formulated an SIRS model with exponential births and deaths, and with potentially increased mortality in both the infected and the recovered classes. In that model, the unique endemic equilibrium for the proportions of individuals in each compartment is globally asymptotically stable.

Here, we have used a simple mathematical model to show that PIM in combination with a return to (some) susceptibility can lead to periodicity. The intuition that underlies this key result is that PIM interferes with reinfection by impacting the secondary susceptible pool. When periodicity occurs, we have shown that the length of the cycles is partly set by the magnitude of the transmission rate (note that larger rates of PIM are needed for larger transmission rates). Furthermore, we found that disease-induced mortality during active infection can synergistically promote cycling. By contrast, robust host immunity (captured via a low relative susceptibility to reinfection) decreases the impact of the secondary susceptible pool on epidemic dynamics, which can therefore cause the disappearance of the cycling behaviour. Thus, for childhood infections that are fully immunizing or nearly so, such as measles, our results show that PIM does not qualitatively change the dynamics (i.e. no potential periodicity). This may explain why childhood disease dynamics are so well understood with their dynamics well described by seasonally forced SIR models. On the other hand, for the dynamics of infectious diseases that do not elicit sterilizing lifelong immunity after recovery, our work highlights an overlooked but likely very common driver of complexity, with cycles emerging. In particular, our findings reveal that ‘hidden’ characteristics of (potentially partially) susceptible individuals, beyond their potential susceptibility to reinfection, can destabilize epidemic dynamics. Untangling these characteristics will requires simple models (for intuition) in conjunction with careful data collection and analyses (for calibration). Our work particularly highlights the need to quantify PIM in infectious disease systems without robust immunity.

Since there is already evidence that SARS-CoV-2 infections can lead to elevated mortality after recovery, our results show that it is important to determine the value of *α*_*S*_ for predictions of future SARS-CoV-2 dynamics. Furthermore, given that low amounts of PIM can trigger periodicity, this phenomenon may be widespread for circulating endemic diseases that potentially contribute to early mortality. To measure PIM, large cohort studies designed to study immuno-epidemiology and evolution for SARS-CoV-2 and beyond (as discussed in [39] for SARS-CoV-2) could include explicit longer-term morbidity and mortality.

Building on our work, there are a number of other potential future avenues that should be investigated. In particular, we have assumed SIS-like dynamics, where individuals are either immune for life, or are immediately (potentially partially) re-susceptible to infection upon recovery. However, the impact of a period of ‘perfect immunity’, e.g. strain-transcending immunity for influenza [40], before a return to (partial) susceptibility (akin to the SIR(S) model [24,25]) should be determined. Similarly, we have ignored the possibility of a short recovery period after acute infection with no (or little) risk of additional mortality, with a subsequent increase in PIM. We have also considered a homogeneous population and ignored age heterogeneities. Since transmission patterns could be different across and among different age groups (e.g. older individuals may participate less in transmission), future work should examine the confluence of PIM and age structure. Since periodicity emerges in our model due to the interaction between elevated mortality and reinfections, we conjecture that either a period of complete immunity, a short recovery period with decreased PIM followed by increased mortality, or age structure (where older, or both younger and older, individuals have an elevated risk of PIM) would still result in cycles, provided that this interaction remains present.

Perhaps more importantly, vaccination can have important epidemiological (e.g. [41]) and evolutionary effects (e.g. [42,43]) on pathogen dynamics. Vaccination can prevent (or decrease the likelihood of) infection via host immune responses. Additionally, as highlighted by SARS-CoV-2, vaccines may also potentially decrease the likelihood of long-term symptoms (i.e. ‘long COVID’) after infection [44–46], and may even decrease long-term symptoms for those that were infected before vaccination [47]. Such a decrease of longer-term symptoms could represent a decrease in PIM. Thus, the impacts of vaccination on epidemic dynamics with PIM should be investigated.

In terms of the host response to infection, we have taken the simplifying assumption that a secondary and beyond infection is equivalent to a primary infection. However, the transmissibility and duration of infection may be different. In particular, if host immunity is present, it is possible that a reinfection is both less transmissible and shorter in duration than a primary infection. Future work should examine the impact of these other forms of immunity on epidemic dynamics with PIM. Relatedly, we have assumed that susceptibility after recovery from secondary infections and beyond is identical to susceptibility after recovery to primary infections. In reality, however, their characteristics may differ. First, due to prolonged damage from multiple infections, PIM could increase with the number of infections. Second, immunity may increasingly become more robust as a host experiences multiple infections. Thus, incorporating multiple classes of susceptibility, with potentially different degrees of PIM, is a fruitful avenue for future research.

While we have formulated a population-level model, a key future direction is to take a cross-scale modelling approach with within-host kinetics (e.g. [48]). For example, such a model may be able to untangle the underlying mechanisms that shape PIM, disease-induced mortality during active infection, and immunity following recovery. A within-host model could also reveal how pathogen loads affect the combination of these characteristics. Other interesting avenues would be to study the potential interplay between PIM and different functional forms for births, or dynamic births as in wildlife populations, and to examine the impact of stochasticity (both as a potential driver and in local extinction). Finally, while we have focused on the long-term behaviour in our model, it would be useful to characterize the transient dynamics (see e.g. [49,50] for transient dynamics in ecology) that emerge due to PIM.

Overall, our results underline the importance of characterizing different degrees of susceptibility in populations in conjunction with the life-history trajectories of individuals as their susceptibility changes. In particular, we have illustrated that small changes in the characteristics of susceptible individuals after they recover (i.e. increased mortality) can result in surprising population-level effects. More generally, our work underlines the use of simple epidemiological models to obtain qualitative insights. Since the inclusion of PIM induces cycles in the simplest model formulation, our work stresses the importance of investigating the impact of PIM in larger, less tractable models of epidemic dynamics. In tandem, our results suggest that empirical studies to quantify PIM are key for predictability.

## Data Availability

Codes are available as electronic supplementary material [51].

## References

[1] Uusküla A et al. 2022 Long-term mortality following SARS-CoV-2 infection: a national cohort study from estonia. Lancet Reg. Health—Eur. **18**, 100394. (10.1016/j.lanepe.2022.100394) 35505834 PMC9051903

[2] Bhaskaran K et al. 2022 Overall and cause-specific hospitalisation and death after COVID-19 hospitalisation in England: a cohort study using linked primary care, secondary care, and death registration data in the opensafely platform. PLoS Med. **19**, e1003871. (10.1371/journal.pmed.1003871) 35077449 PMC8789178

[3] Xu E, Xie Y, Al-Aly Z. 2022 Long-term neurologic outcomes of COVID-19. Nat. Med. **28**, 2406-2415. (10.1038/s41591-022-02001-z) 36138154 PMC9671811

[4] Al-Aly Z, Bowe B, Xie Y. 2022 Long COVID after breakthrough SARS-CoV-2 infection. Nat. Med. **28**, 1461-1467. (10.1038/s41591-022-01840-0) 35614233 PMC9307472

[5] Al-Aly Z, Xie Y, Bowe B. 2021 High-dimensional characterization of post-acute sequelae of COVID-19. Nature **594**, 259-264. (10.1038/s41586-021-03553-9) 33887749

[6] Bengtsson T, Lindstrom M. 2003 Airborne infectious diseases during infancy and mortality in later life in southern Sweden, 1766–1894. Int. J. Epidemiol. **32**, 286-294. (10.1093/ije/dyg061) 12714551

[7] Finch CE, Crimmins EM. 2004 Inflammatory exposure and historical changes in human life-spans. Science **305**, 1736-1739. (10.1126/science.1092556) 15375259

[8] Crimmins EM, Finch CE. 2006 Infection, inflammation, height, and longevity. Proc. Natl Acad. Sci. USA **103**, 498-503. (10.1073/pnas.0501470103) 16387863 PMC1326149

[9] Xie Y, Xu E, Bowe B, Al-Aly Z. 2022 Long-term cardiovascular outcomes of COVID-19. Nat. Med. **28**, 583-590. (10.1038/s41591-022-01689-3) 35132265 PMC8938267

[10] Munoz N, Castellsague X, de Gonzalez AB, Gissmann L. 2006 Chapter 1: HPV in the etiology of human cancer. Vaccine **24**, S1-S10. (10.1016/j.vaccine.2006.05.115) 16949995

[11] Bjornevik K et al. 2022 Longitudinal analysis reveals high prevalence of Epstein–Barr virus associated with multiple sclerosis. Science **375**, 296-301. (10.1126/science.abj8222) 35025605

[12] Mina MJ, Metcalf CJE, de Swart RL, Osterhaus ADME, Grenfell BT. 2015 Long-term measles-induced immunomodulation increases overall childhood infectious disease mortality. Science **348**, 694-699. (10.1126/science.aaa3662) 25954009 PMC4823017

[13] Mina MJ et al. 2019 Measles virus infection diminishes preexisting antibodies that offer protection from other pathogens. Science **366**, 599-606. (10.1126/science.aay6485) 31672891 PMC8590458

[14] Levine KS et al. 2023 Virus exposure and neurodegenerative disease risk across national biobanks. Neuron **111**, 1086-1093.e2. (10.1016/j.neuron.2022.12.029) 36669485 PMC10079561

[15] Humphreys NE, Xu D, Hepworth MR, Liew FY, Grencis RK. 2008 IL-33, a potent inducer of adaptive immunity to intestinal nematodes. J. Immunol. **180**, 2443-2449. (10.4049/jimmunol.180.4.2443) 18250453

[16] Hodzic Z, Schill EM, Bolock AM, Good M. 2017 IL-33 and the intestine: the good, the bad, and the inflammatory. Cytokine **100**, 1-10. (10.1016/j.cyto.2017.06.017) 28687373 PMC5650929

[17] Kuenzig ME, Manuel DG, Donelle J, Benchimol EI. 2020 Life expectancy and health-adjusted life expectancy in people with inflammatory bowel disease. CMAJ **192**, E1394-E1402. (10.1503/cmaj.190976) 33168761 PMC7669301

[18] Graham AL, Schrom EC, Metcalf CJE. 2022 The evolution of powerful yet perilous immune systems. Trends Immunol. **43**, 117-131. (10.1016/j.it.2021.12.002) 34949534 PMC8686020

[19] Anderson RM, May RM. 1979 Population biology of infectious diseases: part I. Nature **280**, 361-367. (10.1038/280361a0) 460412

[20] May RM, Anderson RM. 1979 Population biology of infectious diseases: part II. Nature **280**, 455-461. (10.1038/280455a0) 460424

[21] Gallos LK, Fefferman NH. 2015 The effect of disease-induced mortality on structural network properties. PLoS ONE **10**, e0136704. (10.1371/journal.pone.0136704) 26313926 PMC4552173

[22] Bjørnstad ON, Shea K, Krzywinski M, Altman N. 2020 The SEIRS model for infectious disease dynamics. Nat. Methods **17**, 557-558. (10.1038/s41592-020-0856-2) 32499633

[23] Busenberg S, van den Driessche P. 1990 Analysis of a disease transmission model in a population with varying size. J. Math. Biol. **28**, 257-270. (10.1007/BF00178776) 2332704

[24] Morris SE, Pitzer VE, Viboud C, Metcalf CJE, Bjørnstad ON, Grenfell BT. 2015 Demographic buffering: titrating the effects of birth rate and imperfect immunity on epidemic dynamics. J. R. Soc. Interface **12**, 20141245. (10.1098/rsif.2014.1245) 25589567 PMC4345488

[25] Saad-Roy C.M. et al. 2020 Immune life history, vaccination, and the dynamics of SARS-CoV-2 over the next 5 years. Science **370**, 811-818. (10.1126/science.abd7343) 32958581 PMC7857410

[26] Saad-Roy CM et al. 2021 Epidemiological and evolutionary considerations of SARS-CoV-2 vaccine dosing regimes. Science **372**, 363-370. (10.1126/science.abg8663) 33688062 PMC8128287

[27] Wagner CE et al. 2021 Vaccine nationalism and the dynamics and control of SARS-CoV-2. Science **373**, eabj7364. (10.1126/science.abj7364) 34404735 PMC9835930

[28] Diekmann O, Heesterbeek JAP, Metz JAJ. 1990 On the definition and the computation of the basic reproduction ratio *R*_0_ in models for infectious diseases in heterogeneous populations. J. Math. Biol. **28**, 365-382. (10.1007/BF00178324) 2117040

[29] van den Driessche P, Watmough J. 2002 Reproduction numbers and sub-threshold endemic equilibria for compartmental models of disease transmission. Math. Biosci. **180**, 29-48. (10.1016/S0025-5564(02)00108-6) 12387915

[30] Becker AD, Grenfell B. 2017 tsiR: an R package for time-series susceptible–infected–recovered models of epidemics. PLoS ONE **12**, e0185528. (10.1371/journal.pone.0185528) 28957408 PMC5619791

[31] Pitzer VE et al. 2009 Demographic variability, vaccination, and the spatiotemporal dynamics of rotavirus epidemics. Science **325**, 290-294. (10.1126/science.1172330) 19608910 PMC3010406

[32] Pitzer VE, Viboud C, Alonso WJ, Wilcox T, Metcalf CJ, Steiner CA, Haynes AK, Grenfell BT, Kaderali L. 2015 Environmental drivers of the spatiotemporal dynamics of respiratory syncytial virus in the United States. PLoS Pathog. **11**, e1004591. (10.1371/journal.ppat.1004591) 25569275 PMC4287610

[33] Hethcote HW, Levin SA. 1989 Periodicity in epidemiological models*.* In *Applied mathematical ecology* (eds SA Levin, TG Hallam, LJ Gross), pp. 193–211. Berlin, Heidelberg: Springer.

[34] Liu WM, Levin SA, Iwasa Y. 1986 Influence of nonlinear incidence rates upon the behavior of SIRS epidemiological model. J. Math. Biol. **23**, 187-204. (10.1007/BF00276956) 3958634

[35] Liu WM, Hethcote HW, Levin SA. 1987 Dynamical behavior of epidemiological models with nonlinear incidence rates. J. Math. Biol. **25**, 359-380. (10.1007/BF00277162) 3668394

[36] Hethcote HW, Stech HW, van den Driessche P. 1981 Nonlinear oscillations in epidemic models. SIAM J. Appl. Math. **40**, 1-9. (10.1137/0140001)

[37] Röst G, Tekeli T. 2020 Stability and oscillations of multistage SIS models depend on the number of stages. Appl. Math. Comput. **380**, 125259. (10.1016/j.amc.2020.125259)

[38] Tidbury HJ, Best A, Boots M. 2012 The epidemiological consequences of immune priming. Proc. R. Soc. B **279**, 4505-4512. (10.1098/rspb.2012.1841) PMC3479815 22977154

[39] Saad-Roy CM, Metcalf CJE, Grenfell BT. 2022 Immuno-epidemiology and the predictability of viral evolution. Science **376**, 1161-1162. (10.1126/science.abn9410) 35679395

[40] Ferguson N, Anderson R, Gupta S. 1999 The effect of antibody-dependent enhancement on the transmission dynamics and persistence of multiple-strain pathogens. Proc. Natl Acad. Sci. USA **96**, 790-794. (10.1073/pnas.96.2.790) 9892712 PMC15215

[41] Arinaminpathy N, Riley S, Barclay WS, Saad-Roy C, Grenfell B. 2020 Population implications of the deployment of novel universal vaccines against epidemic and pandemic influenza. J. R. Soc. Interface **17**, 20190879. (10.1098/rsif.2019.0879) 32126190 PMC7115234

[42] Gandon S, Mackinnon MJ, Nee S, Read AF. 2001 Imperfect vaccines and the evolution of pathogen virulence. Nature **414**, 751-756. (10.1038/414751a) 11742400

[43] Read AF et al. 2015 Imperfect vaccination can enhance the transmission of highly virulent pathogens. PLoS Biol. **13**, e1002198. (10.1371/journal.pbio.1002198) 26214839 PMC4516275

[44] Antonelli M et al. 2022 Risk factors and disease profile of post-vaccination SARS-CoV-2 infection in UK users of the COVID Symptom Study app: a prospective, community-based, nested, case–control study. Lancet Infect. Dis. **22**, 43-55. (10.1016/S1473-3099(21)00460-6) 34480857 PMC8409907

[45] Azzolini E, Levi R, Sarti R, Pozzi C, Mollura M, Mantovani A, Rescigno M. 2022 Association between BNT162b2 vaccination and long COVID after infections not requiring hospitalization in health care workers. JAMA **328**, 676-678. (10.1001/jama.2022.11691) 35796131 PMC9250078

[46] Kuodi P et al. 2022 Association between BNT162b2 vaccination and reported incidence of post-COVID-19 symptoms: cross-sectional study 2020–21, Israel. npj Vaccines **7**, 101. (10.1038/s41541-022-00526-5) 36028498 PMC9411827

[47] Ayoubkhani D et al. 2022 Trajectory of long COVID symptoms after COVID-19 vaccination: community based cohort study. BMJ **377**, e069676. (10.1136/bmj-2021-069676) 35584816 PMC9115603

[48] King A, Shrestha S, Harvill E, Bjørnstad O. 2009 Evolution of acute infections and the invasion–persistence trade-off. Am. Nat. **173**, 446-455. (10.1086/597217) 19231966 PMC4101379

[49] Hastings A. 2018 Transient phenomena in ecology. Science **361**, eaat6412. (10.1126/science.aat6412) 30190378

[50] Heggerud C, Abbott K, Hastings A. 2023 Transient dynamics. In *Oxford bibliographies in ecology* (ed. D Gibson). New York, NY: Oxford University Press.

[51] Saad-Roy CM, Levin SA, Grenfell BT, Boots M. 2023 Epidemiological impacts of post-infection mortality. Figshare. (10.6084/m9.figshare.c.6708367) PMC10336371 37434526

